# New Genetic Markers of Skin T-Cell Lymphoma Treatment

**DOI:** 10.3390/genes15030358

**Published:** 2024-03-13

**Authors:** Vladimír Vašků, Petra Fialová, Anna Vašků

**Affiliations:** 1 1st Department of Dermatovenerology, St. Anne’s University Hospital, Faculty of Medicine, Masaryk University, 60200 Brno, Czech Republic; vladimir.vasku@fnusa.cz (V.V.); petra.fialova@fnusa.cz (P.F.); 2 Department of Pathological Physiology, Faculty of Medicine, Masaryk University, 62500 Brno, Czech Republic

**Keywords:** CTCL, CD147, HLA DRB1*1501

## Abstract

Aim: Cutaneous T-cell lymphomas (CTCL) can be described as chronic skin inflammation lesions with the content of malignant T cells and they are considered to be T-cell-mediated skin diseases. CD147 is recognized as a 58-kDa cell surface glycoprotein of the immunoglobulin superfamily; it can induce the synthesis of MMPs (matrix metalloproteinases) on the surface of tumor cells where it was originally identified. It can also function in adjacent tumor fibroblasts using CD147–CD147 interactions. The polymorphism *rs8259* T/A is situated in the untranslated region (3′UTR) of the CD147 gene. HLA DRB1*1501 takes part in the process of presentation and recognition of different antigens to T cells. It can be expressed by antigen-presenting cells—macrophages, dendritic cells, and B cells. The aim of the study is to test genotype–phenotype associations of both polymorphisms including therapy in a large cohort of CTCL patients. Materials and Methods: A final total of 104 CTCL patients were enrolled in the study. For the first remission at the clinic department, they were treated by means of local skin-directed therapy, phototherapy, and systemic therapy. Genomic DNA was isolated from peripheral blood leukocytes. A standard technique using proteinase K was applied. The polymorphisms *rs8259* T/A (CD147 gene) and *rs3135388* (HLA DRB1*1501) were detected through standard PCR-restriction fragment length polymorphism methods. Results: The severity of the disease (patients with parapsoriasis, stages IA and IB, vs patients with stages IIB, IIIA, and IIIB) was associated with the CD147 genotype: the AA variant was 3.38 times more frequent in more severe cases, which reflects the decision on systemic therapy (*p* = 0.02, specificity 0.965). The AA genotype in the CD147 polymorphism was 12 times more frequent in patients who underwent systemic therapy of CTCL compared to those not treated with this therapy (*p* = 0.009, specificity 0.976). The same genotype was also associated with radiotherapy—it was observed 14 times more frequently in patients treated with radiotherapy (*p* = 0.009, specificity 0.959). In patients treated with interferon α therapy, the AA genotype was observed to be 5.85 times more frequent compared to the patients not treated with interferon therapy (*p* = 0.03, specificity 0.963). The HLA DRB1*1501 polymorphism was associated with local skin-directed therapy of CTCL. The CC genotype of the polymorphism was observed to be 3.57 times more frequent in patients treated with local therapy (*p* = 0.008, specificity 0.948). When both polymorphisms had been calculated together, even better results were obtained: the AACC double genotype was 11 times more frequent in patients with severe CTCL (*p* = 0.009, specificity 0.977). The TACT double genotype was associated with local skin-directed therapy (0.09 times lower frequency, *p* = 0.007, sensitivity 0.982). The AACC genotype was 8.9 times more frequent in patients treated by means of systemic therapy (*p* = 0.02, specificity 0.976) and as many as 18.8 times more frequent in patients treated with radiotherapy (*p* = 0.005, specificity 0.969). Thus, the AACC double genotype of CD147 and DRB1*1501 polymorphisms seems to be a clinically highly specific marker of severity, systemic therapy and radiotherapy of patients with T-cell lymphoma. Conclusion: Although genotyping results were not known during the treatment decision and could not modify it, the clinical decision on severity and therapy reflected some aspects of the genetic background of this complicated T-cell-associated disease very well.

## 1. Introduction

Cutaneous T-cell lymphomas (CTCLs) are a complex group of lymphomas that develop in the skin mostly without the presence of extracutaneous disease at the time of diagnosis. CTCL subtypes are variable in terms of their clinical, histological, and molecular characteristics, and they show either a non-severe course or a very progressive course [1]. The etiopathogenetic mechanisms are not yet fully understood. Although some studies have suggested an association between CTCL and the ecotoxicological effects of the environment (radiation, chemicals, and some viral infections), the etiopathogenesis of the disease is not clear so far [2].

The pathophysiology of CTCL is complex. An interaction of genetic (susceptibility genes), epigenetic and environmental factors with immunomodulation effects can be expected to take part in [1].

Diagnosis is based on clinical and pathological signs which are correlated, and the final decision needs an interdisciplinary approach and very often takes a long time. Both less and more severe clinical pictures of CTCL can be distinguished with various clinical characteristics and prognoses. Less severe CTCL [chronic parapsoriasis transforming itself into mycosis fungoides (MF), stage IA, IB, and IIA] mainly involves only the skin. The IIA stage is connected with lymphadenopathy. These stages usually show a less severe clinical course with a better prognosis. In patients with advanced-stage disease (IIB, IIIA, IIIB, IVA, and IVB), the extracutaneous affliction of lymph nodes, viscera, and blood, or transformation to large cells can be observed. Sézary syndrome (SS) is a leukemic form of MF/CTCL in which erythroderma can be developed. It is an aggressive skin T-cell lymphoma with generalized lymphadenopathy and marked by the presence of neoplastic T cells (Sézary cells) in the skin, lymph nodes and peripheral blood. The onset of symptomatology is mostly rapid. Life expectancy following diagnosis is about four years. On the contrary, MF is an indolent low-grade lymphoma with slow progression for many years, but which gradually worsens in its course.

The treatment decision must be made with respect to short-term as well as long-term targets. Taken together, CTCL therapy options comprise skin-directed therapies, such as topical steroids or phototherapy, and systemic therapies including radiotherapy [1]. At the early stage, skin affected by patches and plaques with various morphologic and pathologic findings thereof can be observed. At this stage, skin-directed therapy can be used with a high degree of efficacy for a long time. Patients with refractory or advanced-stage disease develop more severe signs of the disease and have a poorer prognosis, requiring systemic therapy and/or radiotherapy [2]. It is known that the tumor cells are highly sensitive to radiation therapy. Thus, in patients with localized tumor lesions, radiotherapy, especially electron-beam therapy, can lead to good long-term results in treatment. The need for systemic treatment or a combination thereof with skin-directed therapy is absolute in SS. Although a number of treatment protocols are at one’s disposal, they can bring only limited efficacy and, in many cases, durable remission is not achieved [3].

CD147 is a 58-kDa cell-surface glycoprotein of the immunoglobulin superfamily. It is expressed in many cell types including hematopoietic (leukocytes, erythrocytes, and platelets), epithelial, and endothelial cells [4]. 

CD147 is known to be overexpressed in tumor cells. It was found to be associated with the progression of several solid and hematological malignant diseases, including T-cell lymphoma. The anti-CD147 antibody was found to suppress the proliferation of CTCL tumor cell lines through the downregulation of phosphorylated extracellular-regulated kinase 1/2 and Akt [5].

CD147 stimulates MMP production in stromal cells [6]. MMPs take part in degrading the extracellular matrix, which supports the invasion of malignant cells through the basement membrane, and also participate in the metastatic process [7]. According to our results, the associated MMP-2 gene promotor genotype (GGCCTT) is significantly more frequent in CTCL-IA stage patients compared to all other CTCL groups, including parapsoriasis, with high sensitivity and specificity which seems to reflect the importance of genetic variability in MMP genes in the development of CTCL [8]. For this reason, we also suppose a substantial influence of variability in CD147 genes on some phenotypes of CTCL. The *CD147* rs8259T>A variant is in the 3′-untranslated region (3′ UTR) of *CD147* on chromosome 19. The polymorphism is localized in a seed region for microRNA (miR-492) binding. Therefore, variability in the region in 3′UTR can lead to changes in the gene expression. It was found that the T allele of rs8259 was associated with lower expression of CD147 due to alterations in miRNA-492 binding in the region. Moreover, the expression of CD147 from peripheral blood mononuclear cells was observed to be highest in the AA genotype, medium in the AT genotype and lowest in the TT genotype [9].

HLA-DRB1 (6p21.3) codes for a major histocompatibility complex class II cell surface receptor. The tag HLA-DRB1*1501, T allele of the rs3135388 polymorphism was correlated with high expression of DRB1, DRB5 and DQB1 in a Caucasian population. Changes in DR/DQ expression levels related to different alleles seem to have causal effects in different immunopathologies [10]. An association between the HLA-DRB1*1501 genotype and multiple sclerosis (MS) has been confirmed in several studies, including ours [11]. Using a reverse genetic screen coined phenome-wide association study (PheWAS), an association of the rs3135388 genotype (tagging HLA-DRB1*1501) was tested for 4841 phenotypes. Besides a supposed association with multiple sclerosis, association with other phenotypes was found (some type of cirrhosis of the liver, erythematous conditions, benign neoplasms of the thorax, rheumatoid arthritis, juvenile arthritis, Grave’s disease, type 1 diabetes mellitus, systemic lupus erythematosus, and ulcerative colitis), which seems to demonstrate the complex etiologies associated with the HLA-DRB1*1501 loci [12]. Many aspects of chronic inflammation as well as tumorigenesis could participate in CTCL severity/treatment prognosis of the disease. Other HLA genes were confirmed to be involved in susceptibility to CTCL, but also in the prognosis of CTCL patients (HLA-A*24, A*68, A*69, B*35 and DQB1*05:02, or linked genes) [13].

The aim of our study was to analyze genotype–phenotype associations of both polymorphisms including therapy in a large cohort of CTCL patients.

## 2. Materials and Methods

A total of 104 patients with CTCL were included in the study and examined, diagnosed, and treated at the 1st Department of Dermatovenerology of St Anne’s University Hospital Brno, Faculty of Medicine, Masaryk University, and at the Center for treatment of advanced stages of CTCL functioning at the 1st Department of Dermatovenerology from 1993 to 2023. 

CTCL patients were classified according to tumor node metastasis (TNMB) classification for cutaneous T-cell lymphoma [14,15]. 

The CTCL patients were treated according to their clinical state. Topical steroids, a combination of topical steroids with photochemotherapy (PUVA), PUVA alone, photodynamic therapy for residual disease, UVB narrow band therapy, radiotherapy at the Clinic of Radiation Oncology, Masaryk Memorial Institute and Faculty of Medicine, and topical steroids in combination with systemic therapy (interferon α, retinoids, and rexinoids) were applied to them. All patients who were enrolled in the study were in total initial remission of the disease after the chosen treatment protocol.

All patients were genotyped for two chosen polymorphisms. 

The polymorphism *rs8259* T/A is situated in the untranslated region (3′UTR) of the CD147 gene. The genotypes were detected using the PCR-restriction fragment length polymorphism method (RFLP). The primer sequences (forward: 5′-GAGTCCACTCCCAGTGCTTG-3′; reverse: 5′-CTCGTGAAACACTTCAGAAGGAAAAGA-3′) were used. The MboI was chosen for restriction analysis. The AA genotype (137 and 25 bp), AT genotype (162 bp, 137 bp and 25 bp), and TT genotype (162 bp) could be distinguished in 3% agarose gels with ethidium bromide. 

The polymorphism *rs3135388* (HLA DRB1*1501) was also detected with the PCR-RFLP method. The primer sequences (5′ GTA GAG ATC TCC CAA CAA ATCGA- 3′ and 5′ GAG TCG GTC CTG GGG AAT A-3′) together with the ClaI enzyme for restriction analysis were used for detection of the CC (206 bp), TT (186, 20 bp), and CT (206, 186, 20bp) genotypes on 3% agarose gel with ethidium bromide [11].

All the CTCL patients included to the study were successfully genotyped for both polymorphisms. 

This study was approved by the Committee for Ethics of Medical Experiments on Human Subjects, Faculty of Medicine, Masaryk University, Brno (no. 64/93, 1993), and was performed in adherence to the Declaration of Helsinki Guidelines. Participants provided their written informed consent which has been archived.

## 3. Statistical Analysis

The Hardy–Weinberg equilibrium was calculated for each polymorphism using the Χ^2^ test. 

Supposing the unknown dominance of alleles, four modes of gene expressions were employed: dominant/recessive, where major allele carriers were compared with minor allele homozygotes; heterozygote advantage, when heterozygote carriers were compared to both homozygotes, and a co-dominant, additive, gene-dose-based model, where different values were attributed to three genotypes. The Χ^2^ test was used for calculation. Finally, the allelic model was used to compare allelic frequencies between groups. Fisher’s exact test was applied for calculation, and the Bonferroni correction for multiple comparison was used when necessary. For double genotype difference between the compared groups, the corrected Χ^2^ test was used. A calculator for confidence intervals of the odds ratio in an unmatched case control study (hutchon.net, accessed on) was used for the calculation of the odds ratio and confidential interval, and Clinical Calculator 1—VassarStats for the calculation of the sensitivity and specificity of results. 

Statistica software version 14.0 was used for other statistical analyses.

## 4. Results

The severity of the disease (patients with parapsoriasis en plaque, stages IA and IB vs. patients with stages IIB, IIIA, and IIIB) was associated with the CD147 genotype. We observed significant differences in genotype distribution (Pg = 0.02) as well as in allelic frequencies (*p* = 0.03). The AA variant was 7.29 times more frequent at a more severe stage which reflects decisions on systemic therapy (Table 1).

Significant differences in genotype distribution (Pg = 0.003) as well as in allelic frequencies (*p* = 0.01) were found between patients treated with systemic therapy and those who were not. The AA genotype in the CD147 polymorphism was 11.76 times more frequent in patients treated with systemic therapy of CTCL compared to those who were not (Table 2). In CTCL patients treated with interferon α therapy, the AA genotype was observed to be 5.85 times more frequent compared to patients not treated with interferon therapy (Table 2). Borderline significant differences in genotype distribution (Pg = 0.05) and in allelic frequencies (*p* = 0.07) were proved between patients treated with interferon α therapy and those who were not.

The same AA genotype was also associated with radiotherapy—it was observed 13.8 times more frequently in patients with it (Table 2). Highly significant differences in genotype distribution (Pg = 0.001) and in allelic frequencies (*p* = 0.04) were proved between patients treated with interferon α therapy and those who were not.

The HLA DRB1*1501 polymorphism was associated with local skin-directed therapy of CTCL. A significant difference in allelic frequencies (Pa = 0.01) was proved between patients treated with skin-directed therapy and those who were not. The TT genotype was not detected in our patients, which reflects a minor allele frequency of 10% in the population. The genotype distribution is in Hardy–Weinberg equilibrium. The CC genotype of the polymorphism was found to be 3.98 times more frequent in patients treated with local therapy (Table 3).

When both polymorphisms had been calculated together, even stronger results were obtained. When patients with a high severity of CTCL were compared to those with a lower severity of CTCL, a significant difference in double genotype distribution was observed (Pgg = 0.03). The AACC double genotype was 11 times more frequent in patients with severe CTCL compared to those with a less severe form of the disease (Table 4).

The AACC genotype was 8.9 times more frequent in patients treated with systemic therapy (OR = 8.9, Table 5) and as many as 18.6 times more frequent in patients treated with radiotherapy (OR = 18.6, Table 5). The double genotype distributions for systemic therapy (Pgg = 0.02) and radiotherapy (Pgg = 0.004) between patients treated with the therapies and those who were not were significantly different. Thus, the AACC double genotype seems to be a clinically highly specific marker of severity, systemic therapy including interferon and radiotherapy of patients with skin T-cell lymphoma. 

The TACT double genotype was associated with local skin-directed therapy (OR = 0.08, Table 5). Again, a significant difference in double genotype distribution between patients treated and nontreated with skin-directed therapy was found (Pgg = 0.02). 

## 5. Discussion

Our results can hardly be discussed, because we were not able to find articles with comparable results. According to our results, the homozygote genotype AA with the highest expression in the CD147 polymorphism was combined with the HLA DRB1*1501 homozygote with the lowest expression in patients with severe CTCL, which was reflected in the therapy chosen for these patients. 

We were able to find only one publication in which CDA47 and HLA DR expression were associated with therapy. In the study, glaucoma patients were treated with benzalkonium chloride (BAK). Expressions of CD147 as well as of HLA DR were increased in treated patients compared to the control subjects, as proved on conjunctival impression cytology specimens using flow cytometry. Interestingly, a highly significant correlation between CD147 and HLA-DR expressions was found in the study [16]. 

In terms of the mechanisms of CTCL treatment, it is widely known that successful therapy is reached when focused on immunomodulation, even in severe stages of the skin T-cell lymphoma, which is supported by our own opinion. From this point of view, we can discuss the possible participation of our two investigated molecules and their genetic variability in the treatment process of CTCL patients.

CD147 and cyclophilin A (CypA), which can bind to CD147, are overexpressed in tumor cells. CD147 and CypA were found to be overexpressed by tumor cells of MF/SS, and CypA was also expressed by epidermal keratinocytes in MF/SS lesional skin. Serum CypA levels were correlated with disease severity markers in MF/SS patients. Thus, CD147-CypA interactions can contribute to the proliferation of MF/SS tumor cells, and the disruption of CD147-CypA interactions could be seen as a new therapeutic strategy for the treatment of MF/SS [5].

In CTCL, the tumor cells are derived from CD4 (+) CD45RO (+) T memory cells. Early in patch-stage lesions, a small population of malignant T cells that are situated in the epidermis develops. On the contrary, normal T cells are located in the dermis. These infiltrates of normal lymphocytes contain a relatively large proportion of regulatory T cells (Tregs) which are able to suppress both normal infiltrating immune cells and malignant T cells. During the plaque stage, an increased number of malignant T cells as well as of non-malignant T cells can be observed in plaques, but the proportion of Tregs in the benign lymphocytic infiltrates remains constant. In the tumor lesions, a decreasing number of infiltrating non-malignant T cells and decreasing proportion of Tregs in the benign lymphocytic infiltrates can be found. This picture will be followed by a significantly increased number of malignant T in the dermis. In plaque- and patch-stage lesions, a population of the malignant T cells might express low levels of a transcription factor FOXP3. In tumor lesions, FOXP3 expression seems to be almost absent [17].

Regulatory FOXP3+ T cells (Tregs) constitute 5% to 10% of T cells in normal human skin. They are responsible for the development and duration of immunological tolerance. Tregs also participate in the pathogenesis of T-cell lymphomas of the skin (cutaneous T-cell lymphomas—CTCL), skin tumors, and mastocytosis [8].

Using single-cell RNA sequencing and an artificial-intelligence-based algorithm based on it, the expression of *FOXP3* in the skin was found to be the most potent predictor of CTCL stage which has been identified so far [18]. Individual Tregs are not observed to be stable, especially in advanced stages of CTCL [19]. CD147 can participate in this Tregs stability. Tregs with high CD147 expression can effectively inhibit inflammatory responses and maintain FOXP3 stability [20]. Moreover, CD147 is largely recognized as an effective surface target for immune modulation in tumor therapy [21]. However, although CD147 is a known marker of activated Tregs, its underlying mechanisms remain unclear [22].

Treg cells are more resistant to radiation than other lymphocytes, resulting in their preferential increase. In tumor tissue, Treg cells may assist in immune evasion during therapy. Targeting this population may allow for the enhancement of radiotherapeutic benefit through immune modulation [23].

Taken together, CD147 is an important extracellular molecule that regulates the function and stability of Tregs by regulating Foxp3 protein stability. In terms of our results, the variability of the gene coding for CD147 can be related to the individually modified function of CD147 in CTCL patients. 

Similarly, the variability of the HLA DRB1*1501 gene may modify its function during CTCL development and therapy. Until now, HLA-A*24, A*68, A*69, B*35 and DQB1*05:02 were shown to participate in the prognosis of patients with MF [13].

Although genotyping results were not known during the treatment decision and could not modify it, clinical decisions on severity and therapy reflected some aspects of the genetic background of this complicated T-cell associated disease very well. As usual, other similar studies performed on other populations are necessary for a final discussion of our results.

## Figures and Tables

**Table 1:** Severity of CTCL and patient CD147 genotypes.

Severity	CD147 TT	CD147 TA	CD147 AA	MAF	OR (95% CI)	Sensitivity Specificity Power Test	*p*
High (IIB, IIIA, IIIB) (N = 19)	7	8	4	0.73	7.29 (1.48–35.92) for AA in high severity CTCL	0.210	0.02
Low (parapsoriasis, IA, IB, IIA (N = 85)	46	36	3	0.33		0.965	
All (N = 104)	53	44	7	0.39		0.542	

MAF = minor allele frequency. OR = Odds ratio. CI = confidential interval.

**Table 2:** Systemic therapy, interferon therapy, and radiotherapy and CTCL patients with CD147 genotypes.

Systemic Therapy	CD147 TT	CD147 TA	CD147 AA	MAF	OR (95% CI)	Sensitivity Specificity Power Test	*p*
No (N = 82)	45	35	2	0.31	11.76 (2.1–65.79) for AA in patients treated with systemic therapy	0.227	0.004
Yes (N = 22)	8	9	5	0.76		0.976	
All (N = 104)	53	44	7	0.39		0.765	
Interferon α							
No (N = 82)	44	35	3	0.33	5.85 (1.20–28.47) for AA in patients treated with interferon α therapy	0.182	0.03
Yes (N = 22)	9	9	4	0.63		0.963	
All (N = 104)	53	44	7	0.39		0.460	
Radiotherapy							
No (N = 96)	50	42	4	0.35	13.8 (2.41–79.15) for AA in patients treated with radiotherapy	0.375	0.009
Yes (N = 8)	3	2	3	1.00		0.958	
All (N = 104)	53	44	7	0.39		0.954	

MAF = minor allele frequency. OR = Odds ratio. CI = confidential interval.

**Table 3:** Skin-directed therapy and CTCL patients with HLA DRB1*1501 genotypes.

Skin-Directed Therapy	HLA DRB1*1501CC	HLA DRB1*1501CT	MAF	OR (95% CI)	Sensitivity Specificity Power Test	*p*
No (N = 47)	32	15	0.19	3.98 (1.40–11.33) for CC in patients treated with skin-directed therapy	0.895	0.007
Yes (N = 57)	51	6	0.06		0.319	
All (N = 104)	83	21	0.11		0.669	

MAF = minor allele frequency. OR = Odds ratio. CI = confidential interval.

**Table 4:** Severity and CTCL patients with double genotypes.

Severity	TTCC	TACC	AACC	TTCT	TACT	AACT	OR (95% CI)	Sensitivity Specificity Power Test	*p*
High (IIB, IIIA, IIIB)	7	7	4	0	1	0	11.09 (1.86–65.90) for AACC in patients with high CTCL severity	0.211	0.01
Low (parapsoriasis, IA, IB, IIA)	35	28	2	11	8	1		0.976	
All	42	35	6	11	9	1		0.682	

**Table 5:** Systemic therapy, interferon therapy, radiotherapy, and skin-directed therapy and CTCL patients with double genotypes.

Systemic Therapy	TTCC	TACC	AACC	TTCT	TACT	AACT	OR (95% CI)	Sensitivity Specificity Powertest	*p*
No	37	28	2	8	7	0	8.9 (1.51–52.33) for AACC in patients treated with systemic therapy	0.182	0.02
Yes	5	7	4	3	2	1		0.976	
All	42	35	6	11	9	1		0.618	
Interferon α									
No	36	28	2	8	7	1	8.9 (1.51–52.33 for AACC in patients treated with interferon therapy	0.182	0.02
Yes	6	7	4	3	2	0		0.976	
All	42	35	6	11	9	1		0.618	
Radiotherapy									
No	40	34	3	10	8	1	18.6 (2.97–116.64) for AACC in patients treated with radiotherapy	0.375	0.006
Yes	2	1	3	1	1	0		0.976	
All	42	35	6	11	9	1		0.964	
Skin-directed therapy									
No	12	17	3	6	8	1	0.08 (0.01–0.72) for TACT in patients treated with skin-directed therapy	0.982	0.007
Yes	30	18	3	5	1	0		0.170	
All	42	35	6	11	9	1		0.644	

## Data Availability

The data presented in this study are available on request from the corresponding author.
